# Transiently increased serum ferritin is a marker for IgG-mediated murine anaphylaxis

**DOI:** 10.1016/j.jaci.2025.05.016

**Published:** 2025-05-28

**Authors:** Marat V. Khodoun, Fred D. Finkelman

**Affiliations:** a Division of Rheumatology, Allergy and Immunology, Department of Internal Medicine, University of Cincinnati College of Medicine;; b Division of Immunobiology, Cincinnati Children’s Hospital Medical Center, Cincinnati.

**Keywords:** IgE, histamine, macrophage, mast cell, mouse, neutro-phil, shock

## Abstract

**Background:**

Although anaphylaxis is classically mediated by IgE, mast cells (MCs), and FcεRI, mouse experiments and clinical observations demonstrate an alternative pathway mediated by IgG, IgGFc receptors, and several cell types. Objective: We sought to determine whether increased serum ferritin distinguishes IgG-mediated anaphylaxis from IgE-mediated anaphylaxis and to identify the mechanism for its increase. Methods: Passive and active systemic anaphylaxis was induced, respectively, by injecting mice with an IgE or IgG anti-trinitrophenyl mAb and challenged with trinitrophenylovalbumin, or by injecting mice with goat anti-mouse IgD antiserum and challenged with goat IgG. Readouts were hypothermia and hypomobility. Inhibitors were used to investigate cell types and molecules involved. Serum ferritin was quantitated by ELISA.

**Results:**

IgG-mediated, but not IgE-mediated, anaphylaxis induced a severalfold increase in serum ferritin level beginning less than 0.5 hours after antigen challenge and lasting 24 to 36 hours. Polymorphonuclear leukocytes, MCs, and histamine contributed to the ferritin response, but histamine and platelet-activating factor were insufficient to induce the response, which was oxidation-dependent but shock-independent. Small ferritin responses were generated by mouse and human blood cells cultured with immune complexes and/or histamine.

**Conclusions:**

IgG-mediated, but not IgE-mediated, anaphylaxis in mice is associated with a large, rapid, relatively long-lasting increase in serum ferritin that depends on neutrophils, MCs, oxidation, and histamine. Initial data suggest that immune complexes also increase ferritin in humans. Quantitation of serum ferritin should provide a way to detect IgG-mediated anaphylaxis and could help identify the optimal way to detect and treat anaphylaxis.

Antibody-dependent anaphylaxis is classically mediated by IgE, with antigen (Ag) crosslinking of Ag-specific IgE bound to mast cell (MC)-associated and basophil-associated FcεRI initiating a signaling cascade that leads to activation and degranulation of these cells.^1–3^ This releases vasoactive mediators, including histamine, proteases, and preformed cytokines, and induces the synthesis and secretion of additional mediators and cytokines.^2^ However, antibody-dependent anaphylaxis in mice can also be mediated by IgG/Ag complex crosslinking of FcγRs (predominantly, but not exclusively, FcγRIII, the murine analog of human FcγRIIA) on MCs (predominantly connective tissue MCs^4^), which are induced to release histamine, and on macrophages, neutrophils, and basophils, which are induced to release platelet-activating factor (PAF).^3,5^

Although ethical considerations forbid the experiments needed to prove that IgG-mediated anaphylaxis occurs in humans, a considerable body of clinical evidence makes its existence likely. This evidence includes occurrence of anaphylaxis in people who have IgG, but no detectable IgE antibody to the provoking Ag^6^; anaphylaxis in the absence of elevated serum tryptase and positive skin test results^7^; humanized mouse studies in which IgG mediates anaphylaxis in mice that express human instead of murine FcγRs^8^ and in mice that harbor human MCs^4^; and an increased risk of anaphylaxis in patients with intravenous immunoglobulin–infused common variable immunodeficiency who express an FcγRIIA variant that has an increased affinity for IgG.^9^ Because the FcγRs primarily involved in IgG-mediated anaphylaxis bind immuoglobulin with much lower affinity than does FcεRI,^8,10,11^ considerably larger quantities of antibody and Ag are required to induce IgG-mediated rather than IgE-mediated murine anaphylaxis.^12^ Consistent with this, evidence for human IgG-mediated anaphylaxis is restricted to situations in which people are infused or injected with large quantities of foreign Ags, such as therapeutic mAbs, intravenous immunoglobulin (in IgA-deficient individuals), dextran, aprotinin, and von Willebrand factor (in deficient individuals).^6,13–17^

Because of the different isotypes, receptors, and, to some extent, cell types involved in IgG- versus IgE-mediated anaphylaxis, precautions and therapeutic strategies that would be effective in one type of antibody-mediated anaphylaxis might not work in another type. For this reason, it would be useful to identify markers that could differentiate IgG- from IgE-mediated anaphylaxis. In this regard, we have previously shown that decreased FcγRIII expression on macrophages and neutrophils occurs in IgG-mediated murine anaphylaxis, but not IgE-mediated anaphylaxis.^18^ However, our attempts to extend this assay to humans have not been successful (unpublished data), probably because of difficulties comparing receptor expression on peripheral blood cells obtained at different times from the same individuals. Consequently, we considered whether the involvement of macrophages, neutrophils, and basophils in IgG-mediated, but not IgE-mediated, anaphylaxis might lead to increased serum levels of a protein secreted by these cells, but not by MCs. This consideration led us to the iron storage protein ferritin, which is known to be produced by activated macrophages and related cell types as well as activated neutrophils, but has not been reported to be produced by murine MCs.^19^ Although ferritin levels are increased in iron overload disorders and in several inflammatory diseases,^20^ we hypothesized that a rapid increase followed by a relatively sharp decrease in serum ferritin might, in the appropriate clinical situation, occur during IgG-mediated, but not IgE-mediated, anaphylaxis. The results of the studies described herein confirm this hypothesis. In addition, the robust increase in serum ferritin during IgG-mediated anaphylaxis, the longer persistence of this increase than the increases in other anaphylaxis-associated markers, the ease of quantitating serum ferritin by ELISA, and *in vitro* evidence that IgG/Ag complexes induce ferritin production by both mouse and human blood cells suggest that rapid modulation of serum ferritin levels may be a clinically useful assay for the detection of IgG-mediated anaphylaxis.

## METHODS

### Mice

The wild-type BALB/c male and female mice used were 8 to 12 weeks old, unless otherwise noted. The mice were bred and maintained in a specific pathogen-free facility at the Cincinnati Children’s Research Foundation. All experiments were done with the approval of and in accordance with regulatory guidelines and standards set by the Institutional Animal Care and Use Committee of the Cincinnati Children’s Hospital Medical Center.

### mAbs

Hybridomas were obtained from the following sources: IGELa2 (mouse IgE anti-trinitrophenyl [TNP] mAb)^21^ and 1B7.11 (mouse IgG1 anti-TNP mAb)^22^ from the American Type Culture Collection (Rockville, Md); LA1 mouse IgG1 anti-TNP mAb,^22^ a gift from Mike Robson at King’s College (London, United Kingdom); and ACK2 (a rat IgG anti–c-Kit mAb),^23^ a gift from Richard Grencis at University of Manchester (Manchester, United Kingdom). Unless otherwise indicated, hybridomas were grown as ascites in pristane-primed athymic nude mice or BALB/c mice and mAbs were purified from ascites by ammonium sulfate precipitation (25%-50% saturated for all IgGs), followed by DE-52 cation exchange chromatography for the IgG isotypes.^23^ The following were all purchased from Bio-Legend (San Diego, Calif): 1A8 anti-mouse Ly6G mAb, AF488–anti-mouse c-Kit (clone 2B8), phycoerythrin (PE)– and fluorescein isothiocyanate–anti-mouse IgE (clone RME-1), PE–anti-mouse CD49b (clone DX5), AF647–anti-mouse IL-3R (clone 5B11), Peridinin-chlorophyll-protein complex (PerCP)– and PE–anti-mouse CD3 (clone 145–2C11), PerCP– and fluorescein isothiocyanate–anti-mouse Ly6G (clone 1A8), AF488– and PerCP–anti-mouse CD11b (clone M1/70), PE–, PerCP–, and AF647–anti-mouse CD4 (clone GK1.5), allophycocyanin–anti-mouse CCR3 (J073E5), and AF647–anti-mouse F4/80 (clone QA17A29).

### Reagents

TNP conjugated to the carrier protein ovalbumin (OVA) was made as previously described^12^ at a hapten to carrier protein ratio of 22 moles of TNP per mole of OVA, unless otherwise mentioned. The TNP to carrier protein ratio was calculated as previously described.^12^ The H_1_ receptor–specific antihistamine triprolidine (Sigma-Aldrich, St Louis, Mo) was injected intraperitoneally (0.2 mg in 200 μL saline) 30 minutes before TNP-OVA challenge. Albuterol (Sigma) was also injected intraperitoneally (2.5 mg/kg in saline) 30 minutes before TNP-OVA challenge. We purchased heparin sodium (Hospira, Inc, Lake Forest, Ill) and chlodronate and control liposomes (Standard Macrophage Depletion Kit; Encapsula NanoSciences, LLC, Brentwood, Tenn). *N*-Acetylcysteine (Thermo Fisher Scientific, Waltham, Mass) was dissolved in sterile PBS at a concentration of 40 mg/mL and was injected intraperitoneally (400 mg/kg in 0.2 mL saline) twice a day for 3 days.

### IgG/Ag immune complexes

These were produced by mixing equal volumes of saline solutions of 1B7.11 (0.2 mg/mL) with TNP-OVA (0.15 mg/mL).

### *In vitro* cultures

Whole heparinized BALB/c or human blood, mixed with an equal volume of PBS, was cultured at 37°C in 96-well Nunc cell culture plates (Thermo Fisher Scientific, Waltham, Mass) with 0.1 mM histamine and/or immune complexes (ICs). After 6 hours of culture, 4 samples of cultured blood from the same source were pooled and centrifuged, after which supernatants were tested for ferritin concentration by ELISA.

### Passive systemic anaphylaxis

Mice were injected intravenously with mouse IgE anti-TNP or IgG1 anti-TNP mAb, and then challenged intravenously with TNP-OVA 24 or 2 hours later, respectively. Anaphylaxis severity was assessed by measuring rectal temperature with an electronic rectal probe^24^ every 5 to 15 minutes during the 60 to 90 minutes following TNP-OVA challenge. Anaphylactic shock severity varies directly with degree of hypothermia. In some experiments, mice were scored for decreases in mobility,^25^ ranging from 0 (normal mobility) to 5 (death).

### Flow cytometry

Mouse blood was harvested in EDTA tubes, and red blood cells were lysed with RBC Lysis Buffer (BioLegend), washed with PBS, resuspended in Cell Staining Buffer (BioLegend), and stained with fluorochrome-labeled antibodies (BioLegend) according to the manufacturer’s protocol. Peritoneal lavage cells were harvested with PBS supplemented with 10% newborn calf serum (Thermo Fisher Scientific), and red blood cells were depleted with RBC Lysis Buffer, washed with PBS, and stained with fluorochrome-labeled antibodies according to the manufacturer’s protocol (all BioLegend). Flow cytometry was performed with a BD LSRFortessa flow cytometer (BD Biosciences, Franklin Lakes, NJ) and data were analyzed with FlowJo software (FlowJo).

### Ferritin measurement

Serum ferritin levels were measured in mouse plasma obtained by periorbital bleeding 6 hours postchallenge, unless otherwise noted. Ferritin-L levels were measured with the Mouse Ferritin ELISA Kit FTL (AB157713; Abcam, Cambridge, United Kingdom) and ferritin-H/L heteropolymer levels were determined with the Mouse Ferritin ELISA Kit (REF EEL095; Invitrogen/Thermo Fisher Scientific) according to the manufacturers’ protocols. Human ferritin levels were measured with the Human Ferritin ELISA Kit (REF EHFTL; Thermo Fisher Scientific) according to the manufacturer’s protocol.

### Statistical analysis

The nonparametric Mann-Whitney *U* test (GraphPad Prism 10; GraphPad Software, La Jolla, Calif) was used to compare mean maximum temperature drop for groups of mice. For experiments that compared more than 2 groups, the nonparametric Kruskal-Wallis test was used to determine the overall statistical significance of the results; this was followed by comparisons of 2 groups with the Mann-Whitney *U* test. The Kruskal-Wallis test results are shown in figures only when they were not significant. The Pearson test was used to determine *r* and *P* values for correlations. Two-tailed tests were used except when a specific unidirectional hypothesis was being tested (eg, a hypothesis that antihistamine would reduce anaphylaxis severity); in such cases, a 1-tailed test was used. A probability of less than .05 was considered significant.

## RESULTS

### IgG-mediated, but not IgE-mediated, anaphylaxis is accompanied by a substantial increase in serum ferritin

**FIG 1.**
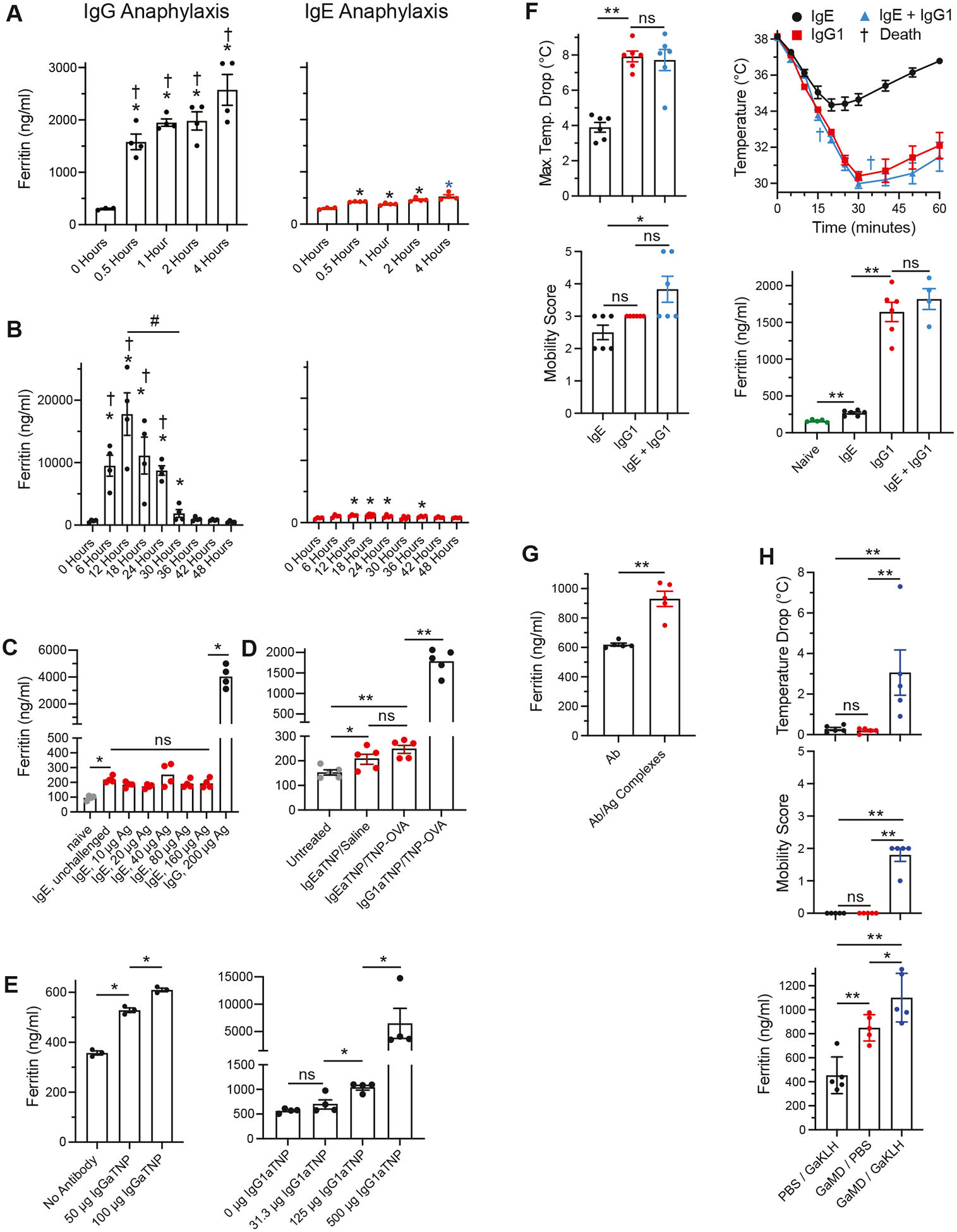
Serum ferritin levels increase during IgG-mediated anaphylaxis. **A** and **B,** BALB/c mice (4/group, mixed male + female, 8–10 weeks old in this and subsequent experiments, unless otherwise noted) were primed intravenously with 500 μg of IgG1 anti-TNP mAb (1B7.11) and challenged intravenously 2 hours later with 200 μg of TNP-OVA, or primed intravenously with 10 μg of IgE anti-TNP mAb and challenged intravenously the next day with 10 μg of TNP-OVA. Mice were bled at the time points after challenge shown. All mice were treated with 20 mg/kg of albuterol intraperitoneally 30 minutes before challenge to suppress shock. Serum ferritin levels were determined with a Thermo Fisher Scientific ELISA kit (Fig 1, A) or an Abcam kit (Fig 1, B). Horizontal lines above bars indicate comparisons made. The 1-tailed Kruskal-Wallis test was followed by the 1-tailed Mann-Whitney *U* test for this and subsequent figures for increase from untreated (0 hour) value (*P < .05) or increase in IgG anaphylaxis value compared with IgE anaphylaxis value at the same time point (†*P* < .05). #*P* < .01. **C,** BALB/c mice were left untreated, primed with 10 μg IgE anti-TNP, and challenged the next day with TNP-OVA (doses shown) or primed with 500 μg of IgG1 anti-TNP and challenged 2 hours later with 200 μg of TNP-OVA. Mice were bled 6 hours postchallenge. Serum ferritin levels were determined with a Thermo Fisher Scientific ELISA kit. **D,** BALB/c mice (5/group) were treated as shown and bled 6 hours later. Serum ferritin levels were determined with a Thermo Fisher Scientific ELISA kit (**P < .01). **E,** In 2 separate experiments, mice were left unprimed or primed with the dose of IgG1 anti-TNP mAb shown and challenged 2 hours later with 100 μg of TNP-OVA (*left panel*) or 200 μg of TNP-OVA (*right panel*) and bled 6 hours (*left panel*) or 12 hours (*right panel*) postchallenge. Serum ferritin levels were determined with a Thermo Fisher Scientific (*left panel*) or an Abcam (*right panel*) ELISA kit. **F**, BALB/c mice were sensitized intravenously with 10 μg of IgE anti-TNP mAb, 500 μg of IgG1 anti-TNP mAb, or both mAbs and challenged intravenously 1 day after IgE sensitization and 2 hours after IgG1 sensitization with 100 μg of TNP-OVA. Mice were followed for 1 hour postchallenge for development of hypothermia and hypomobility and bled 6 hours postchallenge for determination of serum ferritin levels. **G,** BALB/c mice were injected intraperitoneally with saline or premade ICs composed of 0.5 mg IgG1 anti-TNP mAb + 0.05 mg TNP-OVA and bled 6 hours later for determination of serum ferritin levels (Thermo Fisher Scientific ELISA kit). **H,** BALB/c mice were injected intraperitoneally with PBS or 0.2 mL of goat anti-mouse IgD antiserum and challenged intravenously 12 days later with 100 μg of the IgG fraction of goat anti-KLH antibody. Mice were followed for 1 hour for development of hypothermia and hypomobility and bled 6 hours postchallenge for determination of serum ferritin levels. *Ab*, Antibody; *GaKLH*, goat anti-keyhole limpet hemocyanin; *GaMD*, goat anti-mouse IgD; *NS*, not significant.

To determine whether increases in serum ferritin occur during IgG-mediated and/or IgE-mediated anaphylaxis, BALB/c mice were sensitized with IgG1 or IgE anti-TNP mAb, challenged with TNP-OVA, and bled 30 minutes to 48 hours later, after which serum ferritin levels were determined by ELISA. IgG-mediated anaphylaxis was associated with a severalfold increase in serum ferritin concentration that was apparent by 30 minutes after Ag challenge, peaked between 6 and 18 hours postchallenge, was still considerable 24 hours postchallenge, and returned to baseline by 36 hours postchallenge (Fig 1, A and B). In contrast, IgE-mediated anaphylaxis was accompanied by only a small increase in serum ferritin (Fig 1, A and B), which was caused by priming with diluted IgE anti-TNP mAb–containing ascites, rather than anaphylaxis, because it was not significantly affected by Ag challenge (Fig 1, C and D; see also Fig E1 in this article’s Online Repository at www.jacionline.org), even when the dose of Ag challenge was varied over a 16-fold range. IgG-mediated anaphylaxis caused an increase in serum ferritin level even when an IgG antibody dose as low as 50 μg was used for sensitization, although a dose of 500 μg resulted in a much higher ferritin response than a dose of 125 μg (Fig 1, E). Simultaneous induction of IgE- and IgG-mediated anaphylaxis induced a ferritin response similar to that induced by IgG-mediated anaphylaxis alone (Fig 1, F). Preformed IgG/Ag complexes injected intraperitoneally induced a relatively small but statistically significant ferritin response (Fig 1, G). Immunization of mice with goat anti-mouse IgD antiserum, which induces an IgE anti-goat IgG and a much larger IgG anti-goat IgG antibody response with considerable IC formation,^22^ significantly increased serum ferritin levels 12 days later; ferritin levels were further increased when the goat IgG-immune mice were challenged at this time with goat IgG (Fig 1, H).

### Neither shock nor hemocoagulation contributes to the increase in serum ferritin during IgG-mediated anaphylaxis

**FIG 2.**
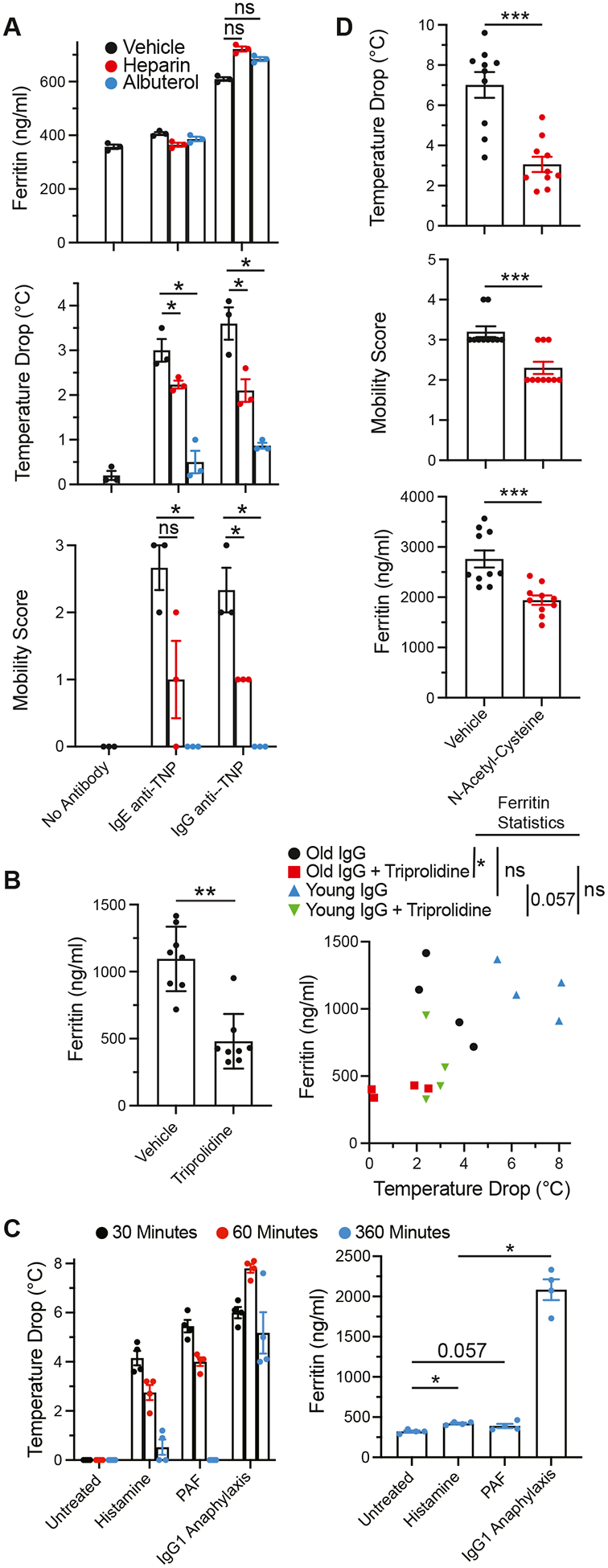
Mechanisms of the increase in serum ferritin level during IgG-mediated anaphylaxis. **A,** Four-month-old BALB/c mice were left unprimed or primed with 100 μg of IgG1 anti-TNP or 10 μg of IgE anti-TNP and challenged with 100 μg of TNP-OVA 1 or 24 hours postpriming, respectively. Mice were bled 2 hours postchallenge. Some mice received 20 IU heparin intravenously or 20 mg/kg albuterol intraperitoneally 30 minutes before challenge. Maximum temperature drop and decreased mobility during the hour postchallenge are shown (Thermo Fisher Scientific ELISA kit). **B,** Eight-week-old (“young”) and 6-month-old (“old”) BALB/c mice were primed intravenously with 125 μg IgG1 anti-TNP, challenged intravenously 2 hours later with 20 μg TNP-OVA, and bled 3 hours after that. Some mice were injected intraperitoneally with 200 μg of triprolidine 30 minutes before challenge. The maximum temperature drop during the hour postchallenge is shown. Serum ferritin level was determined with a Thermo Fisher Scientific ELISA kit. **C,** BALB/c mice were left untreated or injected intravenously with 500 μg of IgG1 anti-TNP. Two hours later, the mAb-injected mice were challenged intravenously with 100 μg TNP-OVA, and some of the previously untreated mice were challenged intravenously with 350 ng PAF or 5 mg histamine. Mice were bled 6 hours after that. Rectal temperatures were determined before challenge and 30, 60, and 360 minutes postchallenge. Serum ferritin level was determined with a Thermo Fisher Scientific ELISA kit. **D,** In 2 separate experiments, BALB/c mice were injected twice a day for 3 days intraperitoneally with vehicle (PBS) or 400 mg/kg *N*-acetylcysteine, and then sensitized intravenously with 0.5 mg IgG1 anti-TNP mAb and challenged intravenously 2 hours later with 100 μg TNP-OVA. Mice were followed for 1 hour for development of hypothermia and hypomobility and bled 6 hours after TNP-OVA challenge for determination of serum ferritin levels (Thermo Fisher Scientific ELISA kit). Values obtained in the 2 experiments were pooled. *NS*, Not significant. *P < .05; **P < .01; ***P < .001; ****P < .0001.

The failure of IgE-mediated anaphylaxis to increase serum ferritin level indicated that shock, by itself, is incapable of increasing serum ferritin, but did not eliminate the possibility that shock contributes to increased serum ferritin during IgG-mediated anaphylaxis. To investigate this possibility, we treated sensitized mice with the β-adrenergic agonist albuterol before Ag challenge and evaluated the effect of this pretreatment on anaphylaxis severity and serum ferritin level. Albuterol pretreatment greatly reduced the hypothermia and hypomobility responses in mice challenged with a relatively low dose of Ag, but had no effect on the increase in serum ferritin (Fig 2, A). The same experiment showed that heparin anticoagulation also failed to affect the increase in serum ferritin level, even though it significantly inhibited hypothermia and hypomobility. The decreased hypothermia associated with IgG-mediated anaphylaxis in this experiment (~4°C) as compared with most other studies of IgG-mediated anaphylaxis in this article (~8°C) is explained by the lower dose of IgG1 anti-TNP mAb used to sensitize mice in this experiment (100 μg as opposed to 500 μg).

### Histamine contributes to serum ferritin response during IgG-mediated anaphylaxis but has little effect on serum ferritin by itself

In contrast to pretreatment with albuterol, antihistamine pretreatment significantly and substantially decreased the serum ferritin response induced by IgG-mediated anaphylaxis (Fig 2, B). This was true in both old and young mice, which developed similar serum ferritin responses even though the younger mice, in this experiment, developed more severe hypothermia than the older mice (Fig 2, B [*right panel*]). However, although intravenous injection of mice with either of the 2 vasoactive mediators that contribute to IgG-mediated anaphylaxis severity (histamine and PAF) induced hypothermia, neither induced more than a small increase in serum ferritin (Fig 2, C). Because reactive oxygen intermediates have been associated with ferritin production,^26^ we evaluated whether pretreating mice with the antioxidant *N*-acetylcysteine would suppress the serum ferritin response to IgG-mediated passive systemic anaphylaxis. *N*-Acetylcysteine pretreatment significantly decreased IgG-mediated anaphylaxis severity and the serum ferritin response, although neither response was totally eliminated (Fig 2, D).

### Increased mouse age is associated with a selective increase in ferritin-L during IgG-mediated anaphylaxis

Ferritin is encoded by 2 genes, one of which (ferritin-H) encodes a larger protein than the other (ferritin-L).^27,28^ Secreted ferritin is composed of 24 ferritin-H and/or ferritin-L monomers, with considerable variability in the ferritin-H to ferritin-L monomer ratio.^20,27^ To evaluate whether age affects the distribution of the ferritin isoforms secreted during IgG-mediated anaphylaxis, we used commercial ELISAs that detect only ferritin-H/ferritin-L or both 6 hours after Ag challenge in 7- and 78-week-old mice. Results showed that although ferritin-H/L levels increased similarly in young and old mice following IgG-mediated anaphylaxis, ferritin-L increased greater than 6-fold more in the older mice (see Fig E2 in this article’s Online Repository at www.jacionline.org). This larger increase in ferritin-L in the older mice was associated with considerably slower recovery from IgG-mediated anaphylaxis in these mice, as determined by return of rectal temperature to normal (Fig E2).

### MCs and neutrophils contribute to the IgG-mediated, anaphylaxis–induced ferritin response

**FIG 3.**
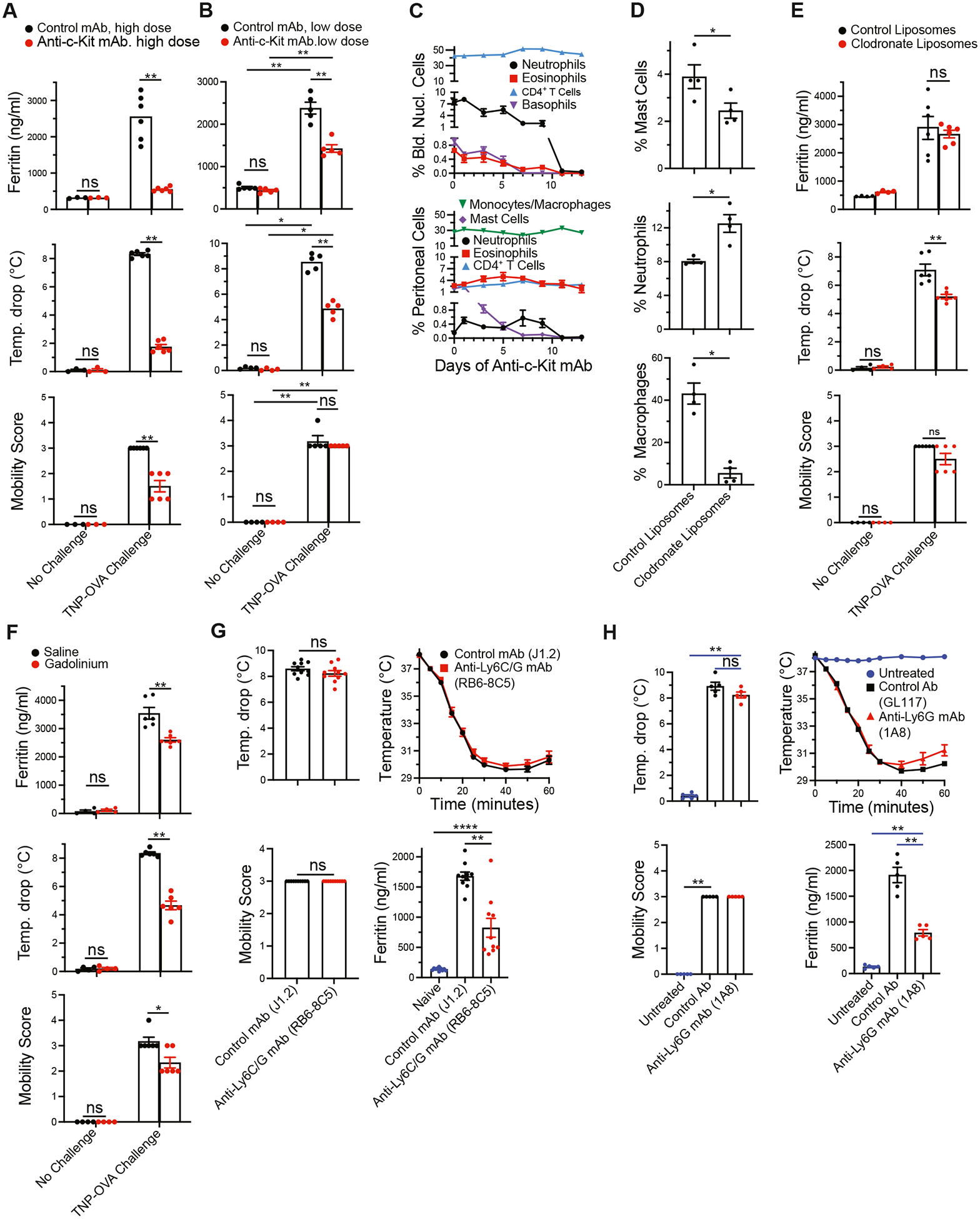
Cells involved in the serum ferritin response to IgG-mediated anaphylaxis. **A,** BALB/c male mice (6/group; 2.5 months old) were injected with 500 μg of mAb ACK2 (rat IgG2b anti–c-Kit) or mAb J1.2 (isotype-matched control) every 2 days for 6 doses (first dose intravenously and subsequent doses intraperitoneally). One day after the last dose, mice were injected intravenously with 500 μg of IgG1 anti-TNP and 2 hours after that intravenously with saline or 100 μg TNP-OVA. Mice were bled 6 hours postchallenge. Maximum temperature decrease and mobility score during the hour postchallenge were determined; serum ferritin levels were determined with a Thermo Fisher Scientific ELISA kit. **B,** Same protocol as in Fig 3, A, except 2-month-old BALB/c mice (mixed sex) received a single injection of 500 μg of ACK2 or J1.2 on day 0 and were primed and challenged on day 7. **C,** BALB/c mice (4/group) were injected intraperitoneally every other day with 0.5 mg of anti–c-Kit mAb or isotype control mAb. Mice were sacrificed 1, 3, 5, 7, 9, 11, or 13 days after the last injection. Percentages of neutrophils, eosinophils, CD4^+^ T cells, and basophils in peripheral blood and percentages of monocyte/macrophages, neutrophils, MCs, eosinophils, and CD4^+^ T cells in peritoneal lavage were determined. **D,** BALB/c mice (4/group) were injected intraperitoneally on days 0 and 5 with clodronate liposomes or control liposomes. Blood nucleated cells obtained on day 6 were stained for CD11b, Ly6G, and CD3; peritoneal lavage cells were partially depleted of adherent cells and stained for CD11b, F4/80, and CD3. **E,** BALB/c male mice (2.5 months old) were injected intraperitoneally on days 0 and 5 with 50 μg of clodronate or control liposomes, primed on day 6 with 500 μg IgG1 anti-TNP, and left unchallenged or challenged 2 hours later with 100 μg TNP-OVA. Mice were bled 6 hours later. Maximum temperature drop and mobility score were determined for the hour postchallenge; serum ferritin levels were determined with a Thermo Fisher Scientific ELISA kit. **F,** BALB/c male mice (2.5 months old) were injected intraperitoneally with saline or with 1 mg of anhydrous gadolinium chloride in 0.2 mL of saline. Mice were primed intravenously with 500 μg IgG1 anti-TNP mAb the next day, left unchallenged or challenged intravenously with 100 μg of TNP-OVA 2 hours later, and bled 6 hours postchallenge. Maximum temperature drop, mobility score, and serum ferritin levels were determined as in Fig 3, E. **G,** BALB/c mice were injected intraperitoneally with 2 mg of RB6–8C5 anti-Ly6C/G mAb or an isotype control mAb. Two days later, these mice were sensitized with 0.5 mg of IgG1 anti-TNP mAb and challenged intravenously 2 hours after that with 100 μg of TNP-OVA. Rectal temperatures and mouse activity were followed for the next hour. Mice were bled 6 hours after Ag challenge for determination of serum ferritin levels with a Thermo Fisher Scientific ELISA kit (data combined from 2 experiments with a total of 10 mice/group). **H,** Same protocol as in Fig 3, G, except the more neutrophil-specific mAb 1A8 anti-Ly6G was used instead of RB6–8C5 anti-Ly6C/G. *Ab*, Antibody; *NS*, not significant. *P < .05; **P < .01; ***P < .001; ****P < .0001.

Because MCs are the source of histamine in murine anaphylaxis^29^ and histamine contributes to the IgG-mediated anaphylaxis–induced ferritin response, even though it is insufficient to induce substantial ferritin production, it seemed likely that MCs contribute to the ferritin response. This hypothesis was tested in experiments that treated mice for different time periods with anti–c-Kit mAb to deplete MCs (Fig 3, A–C). Interpretation of our results was complicated by the different c-Kit dependence of mucosal MCs versus connective tissue MCs on c-Kit (mucosal MCs are rapidly depleted by anti–c-Kit mAb, whereas treatment with this mAb for 5–7 days is required to completely deplete connective tissue MCs^4^) and by the only slightly slower depletion of other cell types that rapidly turn over, such as polymorphonuclear leukocytes (PMLs) (including neutrophils, eosinophils, and basophils) by anti–c-Kit mAb treatment (Fig 3, C). Treatment of mice with multiple doses of anti–c-Kit mAb over 2 weeks almost fully blocked the ferritin response and depleted peritoneal MCs (mostly connective tissue MCs in rodents^30^) but also depleted PMLs (Fig 3, A and C). In contrast, treatment with a single dose of anti–c-Kit mAb for a shorter period of time, which partially suppressed the ferritin response, had little effect on PMLs but partially depleted peritoneal MCs (Fig 3, B and C). The failure of repeated doses of anti–c-Kit mAb over a 2-week period to deplete CD4^+^ T cells or peritoneal macrophages, while it abolishes the ferritin response (Fig 3, A and C), suggests that neither of these cell types contributes substantially to IgG-mediated anaphylaxis–associated ferritin production. The lack of an important role for macrophages is supported by the ability of intraperitoneally injected clodronate liposomes to deplete peritoneal macrophages (Fig 3, D [*lower panel*]) and partially suppress hypothermia (Fig 3, E [*middle panel*]) without suppressing the ferritin response (Fig 3, E [*upper panel*]). However, gadolinium, which suppresses macrophage function,^31–33^ has a moderate inhibitory effect on the ferritin response (Fig 3, F). In contrast, depletion of neutrophils with the anti-Ly6C/G mAb RB6–8C5 or the more neutrophil-specific anti-LyG mAb (1A8)^34^ did not decrease the severity of IgG-mediated anaphylaxis but suppressed the serum ferritin response by more than 50% (Fig 3, G and H).

### IgG/Ag complexes and histamine each increases the ferritin response by cultured mouse and human blood cells

**FIG 4.**
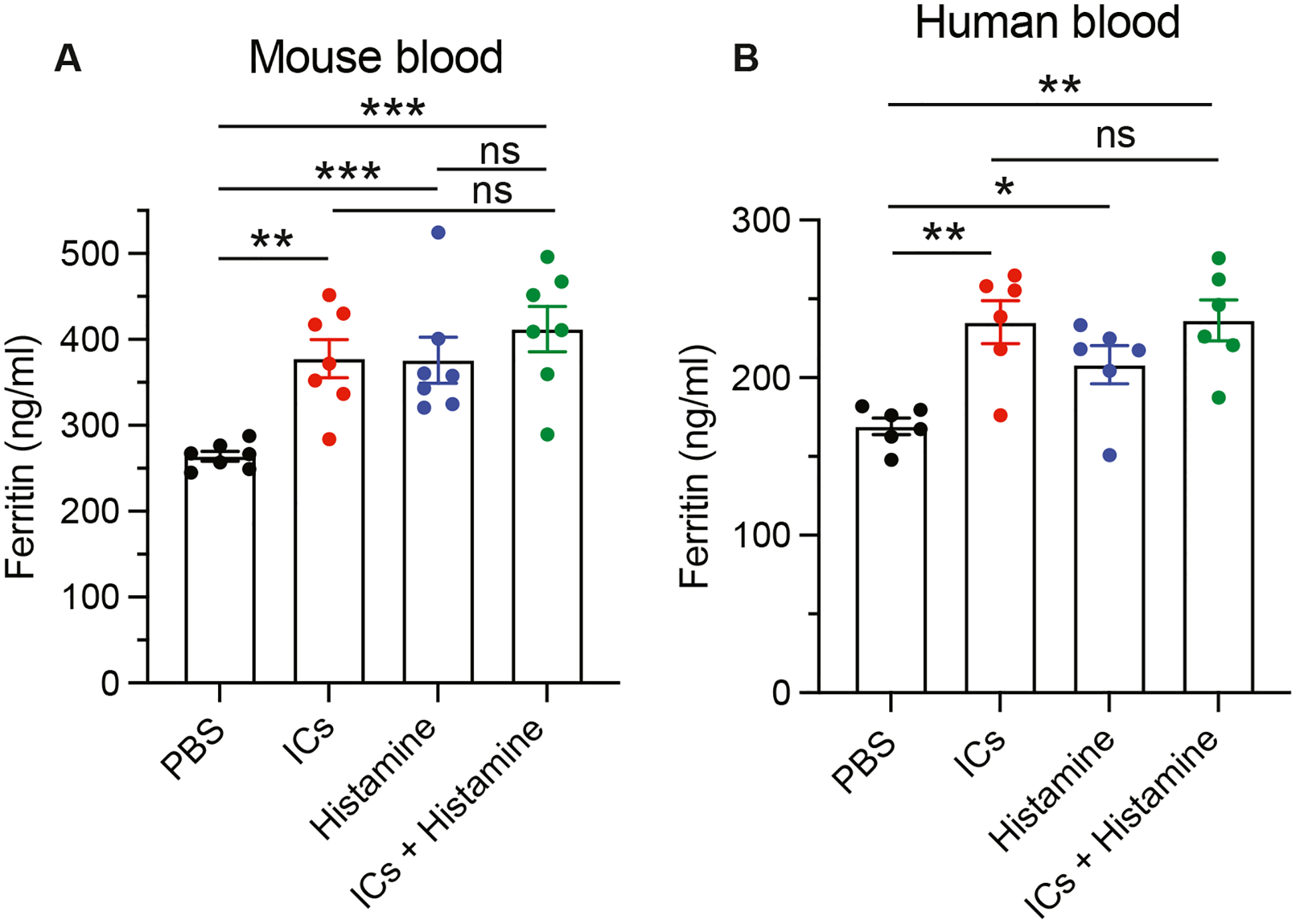
Histamine and ICs increase ferritin release by mouse and human peripheral blood cells *in vitro*. **A,** In 2 separate experiments, 100 μL of whole heparinized blood (4 replicates) from a total of 7 BALB/c mice was cultured for 6 hours with no stimulus, 100 μM histamine, ICs composed of 200 μg/mL of mouse IgG1 anti-TNP mAb + 25 μg/mL of TNP-OVA, or both histamine and ICs, after which 4 technical replicates of culture supernatants from each mouse were pooled and tested for mouse ferritin concentration by an ELISA kit (Thermo Fisher Scientific). **B,** Whole heparinized blood from 3 different individuals (4 replicates) was cultured for 6 hours with no stimulus, 100 μM histamine, ICs composed of 200 μg/mL of mouse IgG1 anti-TNP mAb + 25 μg/mL of TNP-OVA, or both histamine and ICs, after which culture supernatants from each individual were pooled and tested for human ferritin concentration by ELISA. *NS*, Not significant. *P < .05; **P < .01; ***P < .001.

To determine whether our mouse observations about the ferritin response might be applicable to humans, we evaluated the *in vitro* ferritin responses made by mouse and human whole blood cultured with IgG/Ag complexes and/or histamine (Fig 4). IgG/Ag complexes and histamine each induced a small but statistically significant ferritin response after culture for 6 hours. The combined effect of IgG/Ag complexes and histamine was no greater than that of either stimulus alone (Fig 4).

## DISCUSSION

The studies reported herein provide considerable evidence that a rapid, reversible increase in serum ferritin is a useful marker for IgG-mediated murine anaphylaxis that differentiates it from IgE-mediated anaphylaxis, along with preliminary evidence that a ferritin response may also occur in human IgG-mediated anaphylaxis. Serum ferritin levels increase severalfold within 30 minutes of Ag challenge and persist for more than 24 hours but less than 36 hours in IgG-sensitized mice. This period of time makes it convenient to obtain both the serum evidence for a substantial increase in ferritin level and the return to baseline level that are essential to support the diagnosis of IgG-mediated anaphylaxis. This gives serum ferritin quantification an advantage over the measurement of serum levels of other molecules that are associated with anaphylaxis, such as MC-produced histamine and tryptase, that more rapidly return toward normal.^35^ Serum ferritin measurements should also be more convenient and reproducible than flow-cytometric quantification of cellular FcR expression.

A ferritin response can be induced both by IgG/Ag complexes that form *in vivo* and by *in vivo* injection or *in vitro* culture with preformed IgG/Ag complexes (Fig 1, G, and Fig 4, respectively), although the complexes that form *in vivo* induce a considerably greater response. The serum ferritin level is also increased during the immune response to a strong immunogen that is known to be accompanied by IC formation,^22^ and it is further increased during the anaphylactic response invoked by challenge with the relevant Ag (Fig 1, H).

The ferritin response that accompanies IgG-mediated anaphylaxis includes more than 1 species of ferritin, with different ferritin species increased to different extents. Although both ferritin composed solely of 24 ferritin-L monomers and ferritin composed of ferritin-L and ferritin-H increase during IgG-mediated anaphylaxis, the considerable increase in ferritin 24-mers composed only of ferritin-L, especially in older mice, suggests that a ferritin ELISA that can detect both ferritin 24-mers composed only of ferritin-L and ferritin 24-mers that contain both ferritin-L and ferritin-H is preferable to an ELISA that detects only that ferritin that contains both types of monomer. Because no ELISA is available that detects ferritin 24-mers that contain ferritin-H but not ferritin-L, we are unable to tell whether ferritin 24-mers with this composition contribute to the response to IgG-mediated anaphylaxis.

Serum ferritin levels typically increase in response to iron overload disorders, such as hemochromatosis,^36^ and in response to inflammation, especially in adult Still disease, macrophage activation syndrome, severe antiphospholipid antibody syndrome, septic shock, systemic lupus erythematosus, polymyositis/dermatomyositis, severe coronavirus disease 2019 infection, and cytokine storm.^20^ Although a considerably elevated random serum ferritin level would be consistent with any of these disorders in addition to IgG-mediated anaphylaxis, the increase in serum ferritin caused by all of these disorders except IgG-mediated anaphylaxis is prolonged, but the serum ferritin returns to baseline by 36 hours after the onset of IgG-mediated anaphylaxis. Consequently, an observation that serum ferritin is increased in the context of a disorder that has the clinical features of anaphylaxis and returns to normal within 36 to 48 hours should strongly support a diagnosis of IgG-mediated anaphylaxis.

Our observations provide some understanding of the mechanisms responsible for the substantial, reversible increase in serum ferritin that accompanies IgG-mediated murine anaphylaxis, but also reveal complexities that will require additional investigation. The ferritin response to IgG-mediated anaphylaxis is not simply due to shock. Serum ferritin barely increases in response to doses of histamine and PAF that induce substantial hypothermia; is not prevented by drugs, including a β-adrenergic agonist, that strongly inhibit hypothermia; and is not seen with IgE-mediated anaphylaxis. However, although histamine, by itself, has little ability to increase serum ferritin, both histamine and the MCs that produce it contribute substantially to the ferritin response. This suggests that a synergistic response to histamine and additional stimuli is required for IgG-mediated anaphylaxis to induce a maximal increase in serum ferritin levels.

The development of a substantial ferritin response to IgG-mediated, but not IgE-mediated, anaphylaxis implicates cells that express FcγRs, but not FcεRI, and are capable of producing ferritin as the source of the additional stimulus or stimuli. Such cells include macrophages, macrophage-related cells such as Kupffer cells, and PMLs.^19^ Although macrophages, including Kupffer cells, can produce ferritin, peritoneal macrophage and Kupffer cell elimination with intraperitoneally administered clodronate liposomes had no effect on the ferritin response during IgG-mediated anaphylaxis, and the persistence of macrophages after 2 weeks of anti–c-Kit mAb treatment did not prevent loss of the ferritin response. The c-Kit requirement for this response, along with the loss of PMLs concurrent with the loss of the ferritin response, suggests that PMLs contribute to the response along with MCs. Partial suppression of the serum ferritin response by gadolinium, which inhibits macrophage function,^31,32,37^ despite the lack of an effect of intraperitoneal administration of clodronate liposomes, could indicate the importance of a macrophage population that escapes killing by clodronate liposomes administered through this route, but could also reflect the ability of gadolinium to indirectly interfere with PML function.^38^ The importance of PMLs in the ferritin response that is associated with IgG-mediated anaphylaxis is suggested most directly by suppression of the ferritin response by more than 50% by PML depletion by anti-Ly6C/G mAb or the more neutrophil-specific anti-Ly6G mAb, even though treatment with the former mAb fails to suppress IgG-mediated anaphylaxis, as previously reported.^5,39^ We favor neutrophils over other PMLs as the predominant source of ferritin during IgG-mediated murine anaphylaxis because neutrophil numbers considerably exceed the numbers of basophils and eosinophils and because neutrophils, unlike basophils and eosinophils, have been reported to produce ferritin.^19^ The lack of a serum ferritin response in IgE-mediated anaphylaxis, which is MC-mediated, provides additional evidence that MC activation with histamine secretion is insufficient to induce this response. It is also unlikely that the ferritin response is derived from an MC subpopulation that participates in IgG-mediated, but not IgE-mediated, anaphylaxis because our previous studies show that FcγR-responsive MCs are a subpopulation of FcεRI-responsive MCs.^4^

The development of a substantial serum ferritin response less than 30 minutes after an Ag challenge that induces IgG-mediated anaphylaxis indicates that at least the initial ferritin response results from ferritin release rather than new ferritin synthesis, although the 24- to 36-hour duration of the ferritin response suggests that new ferritin synthesis occurs later. It is unlikely that the 24- to 36-hour duration of the serum ferritin response reflects a long serum ferritin half-life, because the half-life of serum ferritin has been reported to be less than 10 minutes in both rats and humans.^40^

The rapid development of the serum ferritin response during IgG-mediated anaphylaxis also suggests that a rapid mechanism connects FcγR stimulation with ferritin release. One possibility is that reactive oxygen intermediates are the connecting factor; these are rapidly produced by neutrophils in response to FcγR crosslinking^41^ and are known to induce ferritin production.^26^ Consistent with this possibility, the ferritin response, along with the severity of IgG-mediated anaphylaxis, was significantly decreased in mice pretreated with the antioxidant *N*-acetylcysteine. The only partial inhibition induced by this agent suggests that other oxidation-independent mechanisms may also be involved in the ferritin response. However, it is also possible that *N*-acetylcysteine, even at the high dose used, only partially neutralized reactive oxygen before it could have a ferritin-inducing effect.

The likely importance of reactive oxygen as a stimulus for ferritin induction by IgG-mediated anaphylaxis could also implicate complement as a stimulus of the ferritin response, because IgG-mediated, but not IgE-mediated, anaphylaxis can rapidly activate complement,^42^ and neutrophil complement receptors can act additively with neutrophil FcγRs to induce reactive oxygen production.^41^ Other known stimuli of increased serum ferritin level, including inflammatory cytokines such as TNF,^27^ are also associated with anaphylaxis,^43^ but would likely take longer than 30 minutes to be produced themselves and then induce ferritin release; however, they might contribute to maintaining the ferritin response for 24 to 36 hours.

The possibility that reactive oxygen is a proximate stimulus for neutrophil ferritin release would also provide an attractive explanation for how MC-produced histamine is important for the ferritin response to IgG-mediated anaphylaxis even though it has little or no ability to induce a ferritin response by itself. Histamine, acting through neutrophil H_1_ histamine receptors, promotes neutrophil reactive oxygen production that has been induced by other stimuli, as shown by studies with histamine and with H_1_ histamine receptor antagonists.^44,45^ Consequently, histamine released in response to activation of MC FcγRs would be expected to increase reactive oxygen responses in FcγR-activated and complement receptor–activated neutrophils, which would in turn increase their release of ferritin.

Taken together, our observations support the hypothesis that the ferritin response associated with IgG-mediated anaphylaxis is derived to a considerable extent from neutrophils that have been stimulated through their FcγRs and complement receptors to produce reactive oxygen. More definitive testing of this hypothesis and determination of how MC-produced histamine contributes to the ferritin response await experiments with mice that have genetic MC and complement or complement receptor deficiencies, as well as *in vivo* studies with complement, reactive oxygen, and cytokine antagonists.

The potential importance of reactive oxygen as a link between neutrophil FcγR and complement receptor ligation during IgG-mediated anaphylaxis and the serum ferritin response also suggests a selective advantage to the ferritin response. Although reactive oxygen helps protect against bacterial pathogens,^27^ an unchecked reactive oxygen response can cause substantial cellular and organ damage.^41^ The ferroxidase activity of ferritin-H subunits,^27^ which can be amplified by ferritin-L subunits,^27^ should limit oxygen free radical production and the potential damage caused by these compounds.

Our observations indicate that a substantial increase in serum ferritin level accompanies IgG-mediated murine anaphylaxis and should be a useful marker for this disorder, differentiating it from IgE-mediated anaphylaxis and other causes of shock. Our observations also provide important initial evidence about the cellular and molecular mechanisms involved in the IgG-mediated anaphylaxis–associated ferritin response. Interestingly, although IgG/Ag complex stimulation of MCs contributes to both anaphylaxis and hyperferritinemia (by inducing histamine release), the macrophage seems to be the IgG/Ag complex–stimulated cell that cooperates with MCs to maximize anaphylaxis severity, whereas the neutrophil appears to be the IgG/Ag complex–stimulated cell that is predominantly responsible for the ferritin response.

We recognize that the impact of our observations is currently limited by the sparce evidence that the serum ferritin response induced by IgG-mediated murine anaphylaxis also occurs in humans. Currently, this is supported only by our observation that IgG/Ag complexes induce similar significant, albeit small, ferritin responses by cultured mouse and human blood cells (Fig 4). We hope, however, that our observations will encourage clinical investigators to evaluate whether a similar increase in serum ferritin level accompanies human anaphylactic responses that are associated with the large Ag infusions that cause IgG-mediated anaphylaxis.

## Supplementary Material

1

## Key messages

- IgG-mediated, but not IgE-mediated, murine systemic anaphylaxis is accompanied by a severalfold increase in serum ferritin level.
- This increase develops within 30 minutes of Ag challenge and lasts for 24 to 36 hours.
- This increase is neutrophil-, MC-, histamine-, and reactive oxygen–dependent.
- ICs and histamine increase ferritin responses by cultured mouse and human blood cells.

## Abbreviations used

Ag: Antigen
IC: Immune complex
MC: Mast cell
OVA: Ovalbumin
PAF: Platelet-activating factor
PE: Phycoerythrin
PerCP: Peridinin-chlorophyll protein complex
PML: Polymorphonuclear leukocyte
TNP: Trinitrophenyl
