# Supplementary material for: Transiently increased serum ferritin is a marker for IgG-mediated murine anaphylaxis
Source: J Allergy Clin Immunol. Author manuscript; Available in PMC 2026 May 27. (PMC13213535; doi:10.1016/j.jaci.2025.05.016)
Supplement: 1 [file NIHMS2176748-supplement-1.pdf]

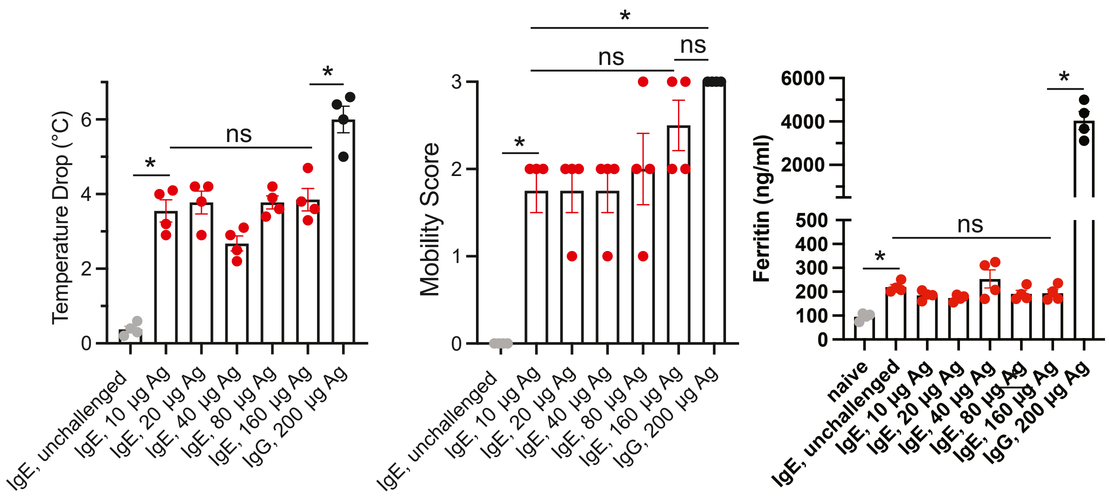

FIG E1. Effect of dose of Ag challenge on severity of IgE-mediated anaphylaxis. Maximum temperature drop and mobility score were determined for 1 hour after Ag challenge for the same experiment shown in Fig 1, *C*. *NS*, Not significant. $*P < .05$.

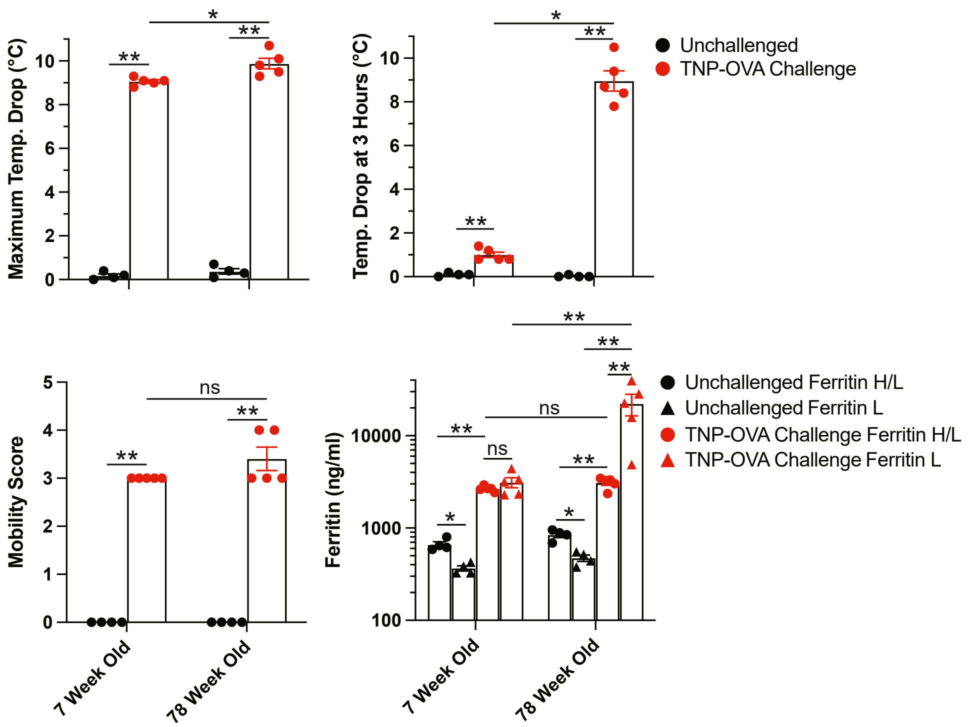

FIG E2. Effect of mouse age on severity and ferritin responses of IgG-mediated anaphylaxis. BALB/c female mice (5/group; 7 and 78 weeks old) were primed intravenously with 500 μg of IgG1 anti-TNP and left unchallenged or challenged intravenously 2 hours later with 100 μg of TNP-OVA. Maximum temperature drop and mobility score were determined during the hour postchallenge, and temperature drop was also determined 3 hours postchallenge. Mice were bled 6 hours postchallenge. Serum ferritin-L levels were determined with an Abcam ELISA kit; serum ferritin-H/L heterodimer levels were determined with a Thermo Fisher Scientific ELISA kit. *NS*, Not significant. $*P < .05$; $**P < .01$.
